# Changes to Cretaceous surface fire behaviour influenced the spread of the early angiosperms

**DOI:** 10.1111/nph.14264

**Published:** 2016-11-07

**Authors:** Claire M. Belcher, Victoria A. Hudspith

**Affiliations:** ^1^ wildFIRE LabHatherly LaboratoriesUniversity of ExeterPrince of Wales RoadExeterEX4 4PSUK

**Keywords:** angiosperm evolution, Cretaceous, fire intensity, fire spread, wildfire

## Abstract

- Angiosperms evolved and diversified during the Cretaceous period. Early angiosperms were short‐stature weedy plants thought to have increased fire frequency and mortality in gymnosperm forest, aiding their own expansion. However, no explorations have considered whether the range of novel fuel types that diversified throughout the Cretaceous also altered fire behaviour, which should link more strongly to mortality than fire frequency alone.
- We measured ignitability and heat of combustion in analogue Cretaceous understorey fuels (conifer litter, ferns, weedy and shrubby angiosperms) and used these data to model palaeofire behaviour.
- Variations in ignition, driven by weedy angiosperms alone, were found to have been a less important feedback to changes in Cretaceous fire activity than previously estimated. Our model estimates suggest that fires in shrub and fern understories had significantly greater fireline intensities than those fuelled by conifer litter or weedy angiosperms, and whilst fern understories supported the most rapid fire spread, angiosperm shrubs delivered the largest amount of heat per unit area.
- The higher fireline intensities predicted by the models led to estimates of enhanced scorch of the gymnosperm canopy and a greater chance of transitioning to crown fires. Therefore, changes in fire behaviour driven by the addition of new Cretaceous fuel groups may have assisted the angiosperm expansion.

## Introduction

The Cretaceous Period saw major diversification in land plants. Previously, conifers (gymnosperms) and ferns (pteridophytes) dominated Earth's ecosystems until flowering plants (angiosperms) appear in the Early Cretaceous fossil record (*c*. 135 Myr ago (Ma); Friis *et al*., 2006). The earliest angiosperms were small, herbaceous weedy forms growing in riparian settings, which then encroached into the understorey of gymnosperm forests (Brodribb & Field, 2010; Royer *et al*., 2010). By the middle Cretaceous they had diversified their form to include shrubs and small trees that grew beneath a forest canopy still dominated by gymnosperms (Crane & Lidgard, 1989; Friis *et al*., 2011). By *c*. 100 Ma angiosperms had risen to be ecologically important and ferns began to diversify. Fern diversification is thought in part to be due to the new environment created by the evolution of angiosperm forest (Schneider *et al*., 2004) in the Late Cretaceous when angiosperms of all forms, including trees, began to dominate many different environments (Friis *et al*., 2011). By the end of the Cretaceous, angiosperms had become the dominant plant group and attained a near ubiquitous distribution across the planet (Crane & Lidgard, 1989).

Bond & Scott (2010) hypothesized that the spread of angiosperms was promoted by the development of novel fire regimes linked to the addition of highly productive (and flammable) plants, which created a positive feedback analogous to the modern day grass–fire cycle. The addition of fast‐growing ‘ruderal’ forms (Taylor & Hickey, 1996; Wing & Boucher, 1998; Brodribb & Field, 2010; Royer *et al*., 2010) is the basis of their argument, where they outline that early angiosperms would have been able to tolerate frequent disturbance, rapidly acquire new fuel, form large cured fuel loads (promoting frequent fires), and outcompete slower growing plants post‐fire (Bond, 1989; Bond & Scott, 2010). They also note that angiosperms were not mere additions to Mesozoic vegetation; they also created new plant communities (Bond & Scott, 2010) and therefore also entirely new fuel structures.

It is well known that vegetation structure and plant traits influence fire behaviour (Schwilk & Caprio, 2011; de Magalhaes & Schwilk, 2012). The structure of fuel in ecosystems strongly influences surface fire spread, which is driven to a large extent by fuel‐bed characteristics such as: fuel load, bulk density, particle size, heat content and moisture (Rothermel, 1972; Scott & Burgan, 2005). Wildfires are also exceedingly sensitive to the level of atmospheric oxygen (Jones & Chaloner, 1991; Belcher & McElwain, 2008; Belcher *et al*., 2010) such that under enhanced levels of atmospheric oxygen, the probability of ignition and fire spread are both increased (Belcher *et al*., 2010; Watson & Lovelock, 2013). Most models and inverse proxy methods suggest a rise in the abundance of oxygen in the Cretaceous atmosphere (e.g. Bergman *et al*., 2004; Glasspool & Scott, 2010; Lenton, 2013) at the time the first angiosperms appeared, to a peak of 1.55‐times present atmospheric levels (PAL) by the time angiosperms had become ecologically important (Bergman *et al*., 2004). Therefore, wildfires would be expected to have been more widespread in the Cretaceous, both according to evolutionary driven changes in fuel and enhancement of atmospheric oxygen.

To date, alterations in fire behaviour encompassing surface rate of spread, fireline intensity and the heat delivered per unit area have not yet been explored for any major evolutionary innovations in plants in the geological past. This hampers our ability to consider evolutionary changes driven by fire because these factors determine the likely type of damage that may occur following a fire, such as whether a surface fire may transition to a crown fire, or how much of a trees crown might be scorched by the heat from the fire's convective column. This is important to understand for the Cretaceous because it determines the possible impact of changes to fire behaviour on gymnosperm forests, and therefore may provide clues as to how small stature plants were able to so rapidly outcompete tall gymnosperm trees to become the most dominant plant group in the world today. In the present study we explore the ability of key Cretaceous surface fuel groups to influence fire behaviour, and consider the feedback of superambient atmospheric oxygen on the projected Cretaceous fire behaviour. We do this by undertaking flammability experiments in analogue Mesozoic fuels, the results of which are used to model Cretaceous surface fire behaviour. Our aim was to consider whether angiosperm additions to key fuel groups and the diversification of ferns in the Late Cretaceous created fire behaviour capable of enhancing gymnosperm mortality and favouring the post‐fire recruitment of angiosperms.

## Materials and Methods

### Flammability experiments in Cretaceous analogue fuels

Twenty‐three plant species were analysed for their ignitability (Table 1), and 22 for their heat of combustion (MJ kg^−1^) (Table 1 and Supporting Information Table S1). These included: extant pteridophytes (ferns and horsetails)*,* weedy dicotyledonous angiosperms and angiosperm shrubs, as well as canopy‐forming conifers that would have provided litter as surface fuels encompassing: needle leaved‐, broad leaved‐, and scale leaved morphotypes (see Table 1). The tree fern *Dicksonia antarctica* was also considered to be a subcanopy fuel that would have contributed to litter. Some of the earliest grasses date back to the Late Cretaceous, however they are considered to have been minor components of ecosystems at this time (Stromberg, 2011), so we did not consider them in flammability trials. All species were tested using an iCone calorimeter (Fire Testing Technology, East Grinstead, UK), in both moist (freshly collected live fuel) and oven‐dried states, and analysed in triplicate. A 3‐cm‐depth fuel bed was created for each species by filling a 368 cm^3^ metal mesh basket according to the natural packing density of the leaves. The calorimeter measured time to ignition and the heat of combustion for each fuel sample. This apparatus is an international standard in experimental fire testing (ISO 5660‐Part 1; ASTM E1354) for the measurement of heat release under controlled laboratory conditions, and is further detailed in Methods S1.

**Table 1:** List of analogue Cretaceous fuels tested and their laboratory‐derived measures of flammability: time to ignition and mean effective heat of combustion

Morphotype	Species	Median time to ignition (s)		Mean effective heat of combustion (dry fuel) (MJ kg^−1^)	
		Fresh	Cured/dry		
Angiosperm shrub	*Hypericum* sp.	76	4.5	15.32	17.19
	*Buxus* sp.	85	7.5	18.47	
	*Drimys winteri*	120	5	17.38	
	*Laurus nobilis*	29	4	17.38	
	*Illicium vernum*	59	6	17.40	
Angiosperm weedy	*Rubus fruticosus*	No ignition – 32 s	3	14.63	14.43
	*Urtica dioica*	No ignition	4	13.26	
	*Piper nigrum*	No ignition	3	14.04	
	*Sacandra chloranthoides*	No ignition	4	15.79	
Pteridophytes	*Asplenium* sp.	No ignition	3	14.22	14.18
	*Pteridium* sp.	No ignition – 51 s	3	12.71	
	*Dryopteris* sp.	No ignition	4	14.62	
	*Dicksonia antarctica*	51	6	15.39	
	*Blechnum tabulare*	No ignition	5	14.18	
	*Equisetum robustum*	No ignition – 35 s	6	13.90	
	*Equisetum sp*.	202.5	11	14.28	
Gymnosperm needle/narrow leaved	*Pinus radiata*	49	6	14.84	16.80
	*Abies koreana*	31	10	Not measured	
	*Sequoia sempervirens*	No ignition – 45 s	6	16.64	
Gymnosperm broad leaved	*Cunninghamia konishii*	81	10	16.82	
	*Podocarpus salignus*	58	6	15.01	
Gymnosperm scale leaved	*Cryptomeria japonica*	81	9	18.69	
	*Thujopsis dolabrata*	38	10	18.80	

The mean effective heat of combustion for each morphotype group was used to numerically describe the heat content in the fuel models.

### Estimating Cretaceous fire behaviour

**Figure 1:**
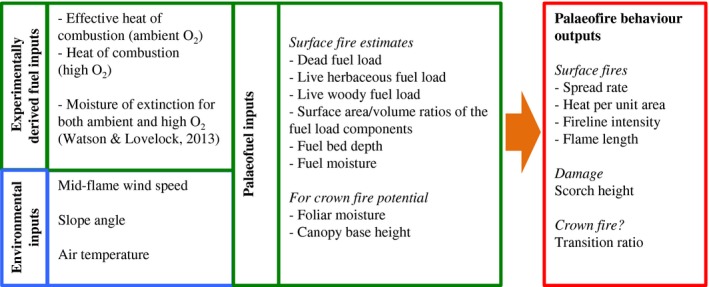
Schematic diagram of the Cretaceous fire behaviour model indicating the inputs required and the outputs generated.

In order to model alterations in Cretaceous fire behaviour, we used the behaveplus modelling system (Andrews, 2009) that is used to predict fire behaviour in modern US ecosystems. We estimated surface fire behaviour using the Surface Module in behaveplus because the new Cretaceous additions were thought to be understorey invaders (Bond & Scott, 2010). behaveplus is a collection of mathematical models that are derived from the Rothermel (1972) fire spread models, which is available as a freeware computer program from https://www.frames.gov/partner-sites/behaveplus/software-manuals/. The behaveplus program is fully detailed in Andrews (2009) and Heinsch & Andrews (2010). A schematic illustrating the components required to build the palaeofire behaviour model (including the input and output parameters) is shown in Fig. 1. behaveplus was used to estimate the following aspects of surface fire behaviour: surface rate of spread (m min^−1^), the heat delivered per unit area (kJ m^−2^), fireline intensity (kW m^−1^) and likely flame lengths (m).

### Construction of Cretaceous fuel models

The fuel in behaveplus is described by a fuel model, which is a set of fuel‐bed parameters that define the amount of fuel available to the fire (fuel load), the bulk density of the fuel and the fuel particle size (leaves, twigs, logs) which are expressed as 1 h, 10 h and 100 h time lag fuels. These are based on the ability of the fuel size class to lose moisture (which determines ignition) and the ‘heat content’ of the fuel (Scott & Burgan, 2005). Fifty‐three pre‐defined standard fuel models exist for use in behaveplus (Scott & Burgan, 2005). In the majority of cases we utilized existing fuel models (Anderson, 1982; Scott & Burgan, 2005) and made minor adjustments to them in terms of heat content and moisture of extinction (explained in the following section). Four fuel models were used to represent key Cretaceous fuel groups; the numeric values that describe these are detailed in Tables 2 and S2. We constructed two fuel models that represented pre‐angiosperm understories: CL for conifer litter and FUn for fern understorey. Two additional models were constructed to represent the early angiosperm understories: WUn for weed‐dominated understorey and SUn for shrub‐dominated understorey. We note that the fern understorey remains relevant throughout the Late Cretaceous as ferns diversified. All fuel models represent the same gymnosperm overstorey populated with either litter, or understorey vegetation with litter. Our fuel models do not relate directly to any specific published Cretaceous fossil flora but are hypothetical explorations of the dominant fuel groups that may have populated the Cretaceous landscape. Fuel model CL is based on the standard fuel model TL3 (Scott & Burgan, 2005) that is a ‘timber litter’ fuel model, where surface fires burn a moderate load of conifer litter that is 9 cm deep, with no understorey. The same forest was then populated with a weedy angiosperm understorey; fuel model WUn is based on the standard fuel model TU3 which has an understorey comprising a moderate amount of forest litter, as well as fine live and dead herbaceous fuel. We defined the herbaceous fuel load to be 0.4 m tall. The same forest was then populated with a shrub understorey, 0.9 m tall to create model SUn, which is based on standard fuel model TU5 and has a heavy forest litter with a high load of shrub understorey. All fuel beds in all models are assumed homogenous and continuous for simplicity, and simulate surface fire behaviour at the flaming front only, and do not take into account any combustion that takes place after the flaming front has passed.

**Table 2:** Numerical description of the four fuel models under ambient oxygen (see Supporting Information Table S2 for superambient oxygen)

Fuel parameter	Fuel model			
	Conifer litter	Fern understorey	Weedy understorey	Shrub understorey
	CL1	FUn1	WUn1	SUn1
1‐h fuel load (t ha^−1^)	1.12	14	2.47	8.97
10‐h fuel load (t ha^−1^)	4.93	4	0.34	8.97
100‐h fuel load (t ha^−1^)	6.28	1	0.56	6.73
Live herbaceous fuel load (t ha^−1^)	0	0	1.46	0
Live woody fuel load (t ha^−1^)	0	1	2.47	6.73
1‐h SA : V (m^2^ m^−3^)	6562	5741	5906	4921
Live herbaceous SA : V (m^−2^ m^−3^)	5906	4921	5249	5906
Live woody SA : V (m^2^ m^−3^)	5249	4921	4593	2461
Fuel bed depth (m)	0.09	0.9	0.4	0.9
Dead fuel moisture of extinction (%)	39	39	39	39
Dead fuel heat content (kJ kg^−1^)^a^	16800	14180	14430	17190
Live fuel heat content (kJ kg^−1^)^a^	16800	14180	14430	17190

a Values are those experimentally derived from our flammability tests.

Ferns, tree ferns, cycads and benettites were typical components of understorey fuels in pre‐angiosperm‐dominated Mesozoic ecosystems (Harris, 1981; Belcher *et al*., 2013). It is likely that ferns were highly important components of surface fuels in pre‐angiosperm ecosystems, and likely retained importance as a fuel load throughout their diversification during the Late Cretaceous (Schneider *et al*., 2004). Ferns grow rapidly and present seasonally dry fuel loads (Agee & Huff, 1987), whereas cycads tend to create minimal litter and have a lower density of fronds. No standard fuel model exists in behaveplus that includes a fern understorey. Fuel model FUn was constructed assuming a dense understorey of ferns. Understories dominated by ferns often form high fuel loads (McDaniel *et al*., 2008; Ainsworth *et al*., 2010), as seen in New Zealand and Hawaii which have fuel loads as high as 70 metric t ha^−1^ (Ainsworth *et al*., 2010), but are more typically of the order of *c*. 20–25 metric t ha^−1^ (McDaniel *et al*., 2008; Pearce *et al*., 2010). We took 20 metric t ha^−1^ as the representative fuel load for our FUn fuel model. This was then apportioned between five fuel classes, as is required for all behaveplus fuel models (Andrews, 2009): 1 h timelag dead fuels which are needles, cured herbaceous and fine dead stems < 0.64 cm; 10 h timelag dead fuels that are cured fuels that are 0.64–2.54 cm diameter; 100 h timelag dead fuels that are 2.54–7.62 cm diameter; live herbaceous fuels, which include grasses and forbs, either annual or perennial; and live woody fuels that are the foliage of very fine stems of living shrubs (Andrews, 2009). In all standard behaveplus fuel models (Scott & Burgan, 2005), live herbaceous fuels are only included in grass containing ecosystems; all other flammable herbaceous fuels (including forbs) are listed as dead and dry/drying. Therefore, to be consistent, we also assumed that all the ferns in the FUn model were dead and thus provided the most flammable fuel load; as such, this model has no live herbaceous fuel load present. Following Anderson's (1982) assignment for timber‐understorey fuel models, we apportioned the majority (70%) of the surface fuel load to the 1 h dead fuel category which is consistent with the dominant size fraction of ferns. Twenty percent of the total load was assigned to the 10 h category, representing thicker dead stems, and 5% to the 100 h category, which could include dead fern bases. Five percent of the load was assigned to the live woody fuel category representing fragments of live fern foliage and rootlets and frond bases that form the stems of tree ferns. Surface area to volume ratios (SA : V, m^2^ m^−3^) for 1 h fuels, live herbaceous and live woody fuels were assigned by selecting the relevant choices (using the choice selection tab) available in behaveplus. Ferns have a lower SA : V than needles and grasses and broad‐leaved trees, and therefore the SA : V ratios for Palmetto understories, the nearest morphological analogue represented currently in behaveplus, were selected as representative. A fuel bed depth of 0.9 m was assigned based on typical understorey fern heights (Ainsworth *et al*., 2010). The heat content used is described in the following section.

All model outputs were compared with existing fuel models and their outputs outlined in Scott & Burgan (2005), to check that sensible values were being estimated, and in the case of the fern fuel model, compared with existing literature (e.g. Ainsworth *et al*., 2010).

### Variable parameters applied within the models

Currently most of the standard fuel models in behaveplus use equal ‘heat content’ for all fuels; however, heat content directly influences fire intensity and different plant materials (particularly shrubs) are known to have variable heat contents (Agee, 1996). Therefore, this aspect of the model is important for our Cretaceous test case because it describes the ability of the surface fuel carrying the fire to release heat energy during combustion. By measuring the effective heat of combustion in our flammability experiments (Table 1) we were able to use ‘heat contents’ (the term used in behaveplus for the effective heat of combustion) that better represented the likely Cretaceous plants described by our fuel models, Tables 1, 2). This approach also allowed us to generate a heat content for our custom fern understorey fuel model that was set to 14 180 kJ kg^−1^ (Table 1).

We do not attempt to capture the full range of surface fire behaviour occurring in the Cretaceous because regional climatic effects, fuel moisture and topographic distributions would have influenced these. However, we have explored a range of environmental effects within the models. When defining a fuel model, the surface fire module of behaveplus requires several environmental inputs: fuel moisture, air temperature, slope angle and mid‐flame wind speed. Slope angle was kept equal between all fuel models and was set assuming a relatively shallow steepness of 10%. Air temperature was set at 25°C for all runs and live fuel moisture was also kept static where we assumed live fuel moisture contents of 100%. This is representative of mature foliage, with new growth complete and is the suggested moisture used for behaveplus when no other information is available. We then ran two experiments, the first that considered variations in dead fuel moisture, which represents the most flammable fuel and a second that explored the sensitivity of the outputs to changes in mid‐flame wind speed (wind speed beneath a forest canopy). Each fuel model was run for four different dead fuel moisture contents of 10, 20, 30 and 60% for a single mid‐flame wind speed of 2.4 km h^−1^, and then over three mid‐flame wind speeds of 2.4, 5 and 5.6 km h^−1^ for a single dead fuel moisture content (10%).

Because the spread of novel fuels during the Cretaceous may have been coincident with enhanced atmospheric oxygen (Bergman *et al*., 2004; Lenton, 2013), we explored its ability to influence fire behaviour. This was represented in the model in two ways: first via altering the moisture of extinction (*M*_ex_) according to the equation *M*_ex_ = 8O_2_–128 (Watson & Lovelock, 2013), whereby enhanced oxygen leads to higher moisture fuels being able to burn. This yields a slightly higher moisture of extinction for present day atmospheric oxygen levels than that used in most standard behaveplus models (Table 2), but using this relationship enabled sensible scaling of moisture of extinction according to changing atmospheric oxygen (Table S2). The slight increase in the ambient oxygen settings value serves only to enhance the point at which the fire will no longer spread, but does not impact the behaviour of the fires themselves. We assumed 26% atmospheric oxygen to be the superambient level in all fuel models (Lenton, 2013). Second, the heat of combustion in the fuel models was increased to reflect potential high Cretaceous atmospheric oxygen. In order to do this we compared the effective heat of combustion derived from our ambient oxygen laboratory experiments (Table 1) with the heat of combustion from published bomb calorimetry data (de Dios Rivera *et al*., 2012) (Table S3), which assesses heat content in a high oxygen atmosphere. We found that the heat of combustion from bomb calorimetry was *c*. 22% higher than in ambient conditions (Table S3). Each fuel model was run twice: the first run assuming ambient/Jurassic atmospheric oxygen (outputs denoted: CL1, FUn1, WUn1 and SUn1) and the second assuming high/late Cretaceous atmospheric oxygen (outputs denoted: CL2, FUn2, WUn2 and SUn2). Table S2 shows the final values of heat content used for the two model types.

### Estimating canopy scorch and potential to transition to a crown fire

In order to consider whether changes in surface fire behaviour may have affected the gymnosperm overstorey, we used the Scorch and Transition to Crown Fire functions of behaveplus to consider the potential height of canopy scorch and whether fires in the various surface fuels may have had the potential to transition to a crown fire.

Scorch height in behaveplus is the height above the ground at which the temperature in the fire's convective column reaches the lethal temperature required to kill live crown foliage (60°C). The two input variables required to determine scorch height, which were derived from the surface module, are flame length and mid‐flame wind speed. Scorch height estimates were made over the four fuel moistures described earlier assuming a wind speed of 2.4 km h^−1^, and then separately for the three wind speeds described earlier, with a dead fuel moisture of 10%.

The Crown function in behaveplus can be used to estimate a transition ratio, which indicates whether a surface fire may transition to a crown fire. It is derived by dividing the surface fireline intensity by the critical surface intensity, which is calculated using canopy foliar moisture and canopy base height. This yields a dimensionless number where values greater than or equal to 1 indicate that the surface fireline intensity is sufficient to cause the surface fire to transition to a crown fire. In order to consider the transition potential, two further inputs were required in the model: an estimate of canopy foliar moisture, and an estimate of canopy base height. Canopy foliar moisture was set to 100% according to Scott & Reinhardt's (2001) suggestion that where unknown 100% is a reasonable estimate for use in behaveplus. Because our fuel group explorations do not represent specific Cretaceous ecosystems and because estimates of tree morphology (tree height and canopy base height) are not easy to estimate from the plant fossil record, we ran the crown function for a range of canopy base heights. The transition ratio was estimated for base heights of 2, 4, 6, 8 12, 16 and 20 m assuming dead fuel moisture set at 10% with the mid‐flame wind speed set at 2.4 km h^−1^. A range of mid‐flame wind speeds were also tested (2.4, 5 and 5.6 km h^−1^), assuming a canopy base height of 6 m, and a dead fuel moisture of 10%.

## Results

### Ignitability of fuels

**Figure 2:**
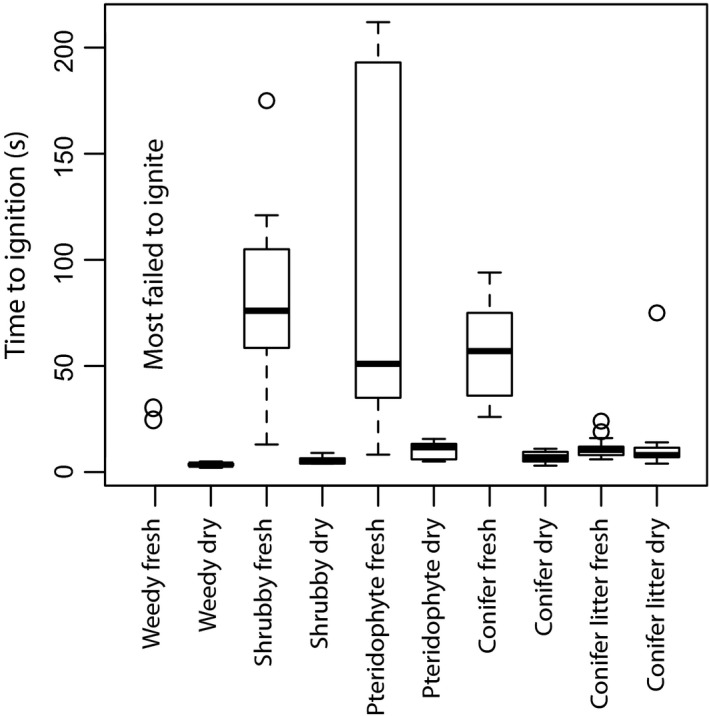
Box plots indicating the time taken for each Cretaceous analogue fuel group to ignite when exposed to a heat flux of 50 kW m^−2^ in an iCone Calorimeter. Results are shown for fresh, moist fuel and fully cured, dry fuel for each fuel group.

Of the fresh fuels tested, gymnosperm litter and pteridophyte litter were the fastest to ignite (Fig. 2; Table 1). The weedy angiosperms failed to ignite in fresh state except *Rubus fruticosus* of which two of the three samples tested ignited after heating *c*. 30 s (Table 1). Both fresh shrubby angiosperms and fresh pteridophytes were ignitable. Once cured, however, all fuels had a similar ignition potential (Fig. 2; Table 1), and despite being the least ignitable when fresh, weedy angiosperms ignited the most rapidly when dry (supporting assertions of Bond & Scott, 2010). However, several species of pteridophyte were observed to have equally rapid ignition. The gymnosperm litter was found to be the most consistently ignitable fuel under a range of conditions (moist or cured; Fig. 2; Table 1); therefore, ignition in conifer litter need not be restricted to the dry season, as is also observed in modern wildfires (Bond & van Wilgren, 1996).

### Model estimates of fire behaviour according to fuel type and dead fuel moisture content in ambient oxygen

**Figure 3:**
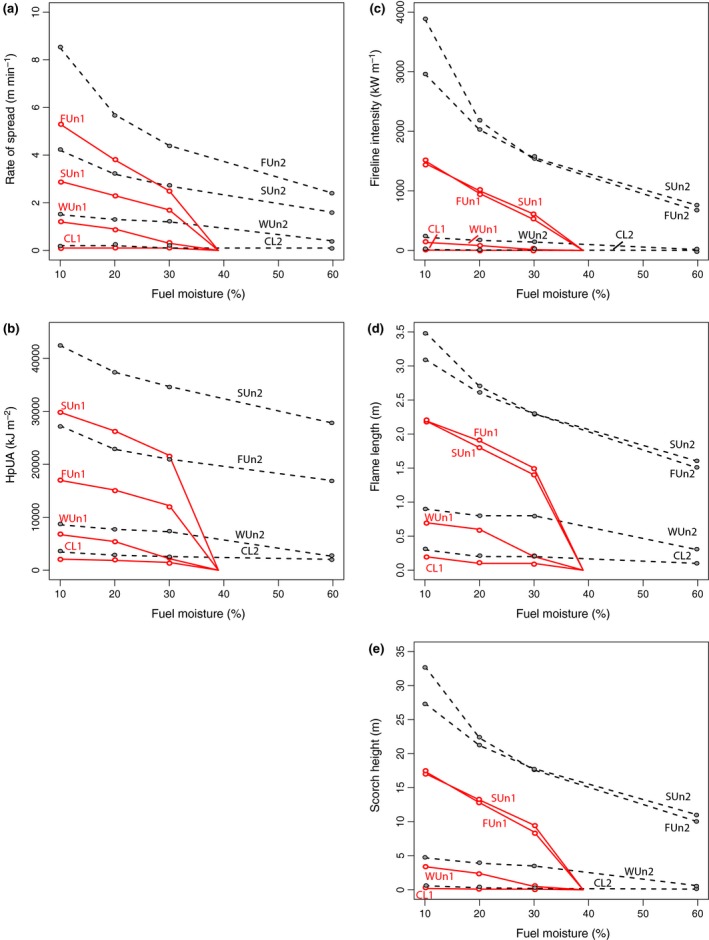
Estimated fire behaviour for the four Cretaceous fuel models according to variations in dead fuel moisture content, showing (a) rate of spread, (b) heat per unit area (HpUA), (c) fireline intensity, (d) flame length and (e) scorch height. Solid red lines with open red circles indicate model outputs for ambient atmospheric oxygen levels (models appended with 1), and dashed black lines with closed grey circles indicate outputs for superambient oxygen (models appended with 2). All models assume a mid‐flame wind speed of 2.4 km h^−1^. Fires cease to spread in ambient oxygen in the models at a moisture of extinction of 39%, hence the decline in the models at that point on the *x*‐axis. Model acronyms: CL, conifer litter; FUn, fern understorey; WUn, weed‐dominated understorey; SUn, shrub‐dominated understorey.

Of the fuel models explored under ambient oxygen conditions, the fern understorey model (FUn) showed the highest rate of surface spread, followed by the shrub understorey (SUn), then the weedy understorey (WUn1) (Fig. 3a). The surface rate of spread in these fuel models was influenced by moisture content, with spread rates declining as dead fuel moisture content increased, until the moisture of extinction was reached in the model (39%) (Fig. 3a). The conifer litter fuel model (CL) had the lowest rate of surface spread and was also much less influenced by differences in moisture content.

The shrub understorey delivered almost double the amount of heat per unit area than any of other understorey fuels, with the fern understorey being the next highest, and conifer litter the lowest (Fig. 3b). All estimates of heat per unit area declined with increasing dead fuel moisture content (Fig. 3b). Both fuel models SUn and FUn generated similar estimates of fireline intensity and were at least 10 times more intense than fires fuelled by conifer needle litter or weedy angiosperms (Fig. 3c). Increasing dead fuel moisture content also significantly decreased the fireline intensity, particularly in the models that yielded high fireline intensity estimates (Fig. 3c). Angiosperm shrub fuels generated slightly lower fireline intensities at low fuel moisture but were able to maintain greater fireline intensities at higher moisture contents. Because flame length is related to fireline intensity, SUn and FUn produced similar flame length estimates (Fig. 3d). Estimates of flame length for WUn were around half those of SUn and FUn, and CL produced flame lengths around half that of WUn. Flame length was similarly influenced by changes in dead fuel moisture content (Fig. 3d).

We also considered the potential height of canopy scorch because this relates to tree mortality (Ryan & Reinhardt, 1988) (Fig. 3e). Fires carried in the fern and shrub understories had a comparable ability to induce canopy scorch tens of metres into the overstorey vegetation. Weedy angiosperm fuels may have induced canopy scorch a few metres in the canopy, whilst conifer litter had limited ability induce any canopy scorch. By considering flame lengths and canopy scorch for the lowest dead fuel moisture (10%), and a single mid‐flame wind speed (2.4 km h^−1^), we estimated the potential ability of the surface fires to transition to a crown fire for a range of canopy base heights. Table 3 shows the transition ratio for each fuel model over a range of canopy base heights. Both the shrub and fern understories were found to have potential to transition to crown fires assuming canopy base heights 4 m or lower.

**Table 3:** Transition ratio for different canopy base heights

Fuel model	Canopy base height (m)						
	2	4	6	8	12	16	20
CL1	0.01	0	0	0	0	0	0
CL2	0.03	0.01	0.01	0	0	0	0
WUn1	0.28	0.1	0.05	0.04	0.02	0.01	0.01
WUn2	0.46	0.16	0.09	0.06	0.03	0.02	0.01
SUn1	3.09	1.09	0.59	0.39	0.21	0.14	0.1
SUn2	6.25	2.21	1.2	0.78	0.43	0.28	0.2
FUn1	3.18	1.12	0.61	0.4	0.22	0.14	0.1
FUn2	8.19	2.9	1.58	1.02	0.56	0.36	0.26

Numbers ≥ 1 suggest that a surface fire may transition to a crown fire. Grey shading highlights surface fires that could transition to a crown fire.

Model acronyms: CL, conifer litter; FUn, fern understorey; WUn, weed‐dominated understorey; SUn, shrub‐dominated understorey; 1 indicates ambient and 2 indicates superambient oxygen levels.

### Sensitivity of the fire behaviour estimates to changes in mid‐flame wind speed (ambient oxygen)

**Figure 4:**
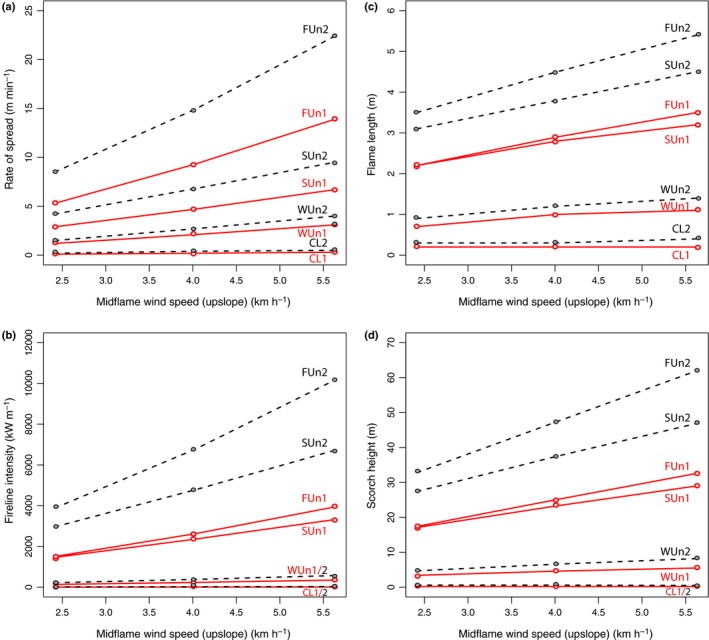
Estimated fire behaviour for the four Cretaceous fuel models according to variations in mid‐flame wind speed. (a) Rate of spread, (b) fireline intensity, (c) flame length and (d) scorch height. All models assume a dead fuel moisture of 10%. Solid red lines with open red circles indicate model outputs for ambient atmospheric oxygen levels, and dashed black lines with closed grey circles indicate outputs for superambient oxygen. Model acronyms: CL, conifer litter; FUn, fern understorey; WUn, weed‐dominated understorey; SUn, shrub‐dominated understorey.

In order to explore how different wind speeds might influence the results, we ran the fuel models for the lowest dead fuel moisture tested (10%), and explored a range of mid‐flame wind speeds (2.4, 4 and 5.6 km h^−1^). Increasing the wind speed increased the surface rate of fire spread (Fig. 4a) but did not alter the overall differences between the fuel models. Fireline intensity was similarly enhanced by increasing the mid‐flame wind speed for all fuel models; however, whereas the fern and shrub understories had appeared to generate similar fireline intensities at wind speeds of 2.4 km h^−1^, the fireline intensity of FUn was shown to increase more than that of SUn as wind speed was increased (Fig. 4b). This therefore had a similar influence on flame length estimates (Fig. 4c). Scorch height was increased in fuel models WUn, SUn and FUn but not in the CL model (Fig. 4d) under increasing wind speeds. The weedy understorey in particular became capable of more significant canopy scorch (up to 5.5 m height) as wind speed increased. Both the fern and shrub understories had the potential to lead to crowning assuming a mid‐flame wind speed of 2.4 km h^−1^ for crown base heights 4 m or lower (Table 4). Increasing mid‐flame wind speed to 4 km h^−1^ increased the height at which model FUn had the potential to transition to a crown fire, enabling transition in canopy base heights up to 6 m; model SUn was similar, with a transition ratio of 0.96 for a 6 m canopy base height.

**Table 4:** Transition ratio for different mid‐flame wind speeds based on a canopy base height of 6 m

Fuel model	Mid‐flame wind speed (km h^−1^)		
	2.4	4.0	5.6
CL1	0	0	0
CL2	0.01	0.01	0.01
WUn1	0.05	0.1	0.14
WUn2	0.09	0.16	0.23
SUn1	0.59	0.96	1.35
SUn2	1.2	1.94	2.73
FUn1	0.61	1.06	1.6
FUn2	1.58	2.75	4.13

Numbers ≥ 1 suggest that a surface fire may transition to a crown fire. Grey shading highlights surface fires that could transition to a crown fire. Model acronyms: CL, conifer litter; FUn, fern understorey; WUn, weed‐dominated understorey; SUn, shrub‐dominated understorey; 1 indicates ambient and 2 indicates superambient oxygen levels.

### Influence of potential superambient oxygen on Cretaceous fire behaviour

All aspects of fire behaviour were enhanced by assuming increased levels of atmospheric oxygen (see dashed lines on Figs 3, 4). The least affected fuel type was conifer litter, which showed relatively small changes in the rate of spread, heat per unit area, fireline intensity and flame lengths. However, in fuel types that were capable of carrying intense fires (fern and shrub understories), enhanced oxygen levels significantly increased the intensity of the fires (Figs 3, 4). This led to the ability to cause scorch at greater heights (Fig. 3) and transition to crown fires in higher canopy base heights (Tables 3, 4).

## Discussion

### Fuel driven changes to Cretaceous fire behaviour

Bond & Scott (2010) argued that early angiosperms required an external factor to escape the shade of forest trees, and suggested that the addition of a new highly flammable fuel (weedy angiosperms) created an angiosperm–fire cycle similar to that of the modern day grass–fire cycle. We found fresh angiosperm fuels were relatively unignitable, but in cured state ignited more rapidly than any other fuel group tested (Fig. 2; Table 1). Therefore, Bond & Scott's (2010) suggestion that weedy angiosperms provided an easily curable and ignitable fuel during the Cretaceous is in part supported by our ignition experiments. However, overall only a small variation in ignitability was observed between all the cured fuels tested and, more significantly, all other plant groups were more ignitable than weedy angiosperm fuels when moist. This suggests that variations in ignition driven by angiosperm additions alone are less likely to have been a significant feedback to changes in Cretaceous fire activity than estimated previously. In modern forests the addition of herbaceous and shrubby angiosperms has been observed to add a significant fine fuel load to otherwise moist forests (Agee, 1996), increasing their fire risk. Therefore, changes in fuel structure and their influence on fire behaviour may have been more important than changes to ignition properties alone. Our models suggest that ecosystems with weedy angiosperm understories would have been capable of carrying more intense and faster spreading fires than those previously dominated by conifer litter (Figs  3, 4). However, the fires would have been unlikely to lead to significant canopy scorch or crown fire activity (Figs 3e and 4d; Tables 3, 4). Therefore, the addition of weedy fuels is unlikely to have rivalled the flammability of ecosystems with pre‐existing fern understories, which we estimate were able to carry significantly more intense and rapidly spreading fires (Figs 3a and 4a).

By the mid‐Cretaceous, angiosperms were present as small trees and shrubs (Friis *et al*., 2011) and from *c*. 100 Myr ago (Ma) ferns also began to diversify (Schneider *et al*., 2004). Our shrub‐dominated understorey (SUn) model suggests that as angiosperm shrubs invaded forest understories they would have carried particularly intense fires (Figs 3, 4). Both the shrub (SUn) and fern (FUn) dominated understory models models generated fireline intensities at least 10 times greater than those fuelled by conifer litter (CL) or weedy angiosperm understorey (WUn) (Fig. 3c). Our models suggest that dense fern understories may have been able to carry the fastest moving fires (Figs 3a and 4a) consistent with observations in modern forests, where ferns can generate large cured surface fuel loads in the dry season, for example dried dead fronds of *Pteridium aquilinum* produce several tons of ‘flashy’ (rapidly burning intense fires) fuel seasonally in the Pacific Northwest of the USA (McCulloch, 1942). While dense fern understories may have continued to carry intense, fast‐moving fires, our models indicate that fires in angiosperm shrub understories were likely to have been capable of delivering twice as much heat per unit area compared with an understorey of ferns (Fig. 3b). This is significant because fire severity typically relates to the duration over which heat from a fire is delivered, where slower moving but more intense fires have the potential to be more ecologically damaging (Belcher, 2016). Moreover, whilst our models suggest that angiosperm shrubs generated slightly lower fireline intensities than ferns at very low fuel moistures, shrubs maintained greater fireline intensity than ferns at higher moisture contents. As such, invasion of angiosperm shrubs into forest understories during the mid‐Cretaceous may have fuelled more ecologically destructive fires, whereas the diversification of ferns in the Late Cretaceous may have ensured rapid fire spread and maintained the importance of fire in fern understories. This hypothesis is supported by an increase in the occurrence of charcoal in the fossil record, across a range of Cretaceous environments from peatland/mires through to floodplains (Belcher & McElwain, 2008; Bond & Scott, 2010; Glasspool & Scott, 2010). These fossil charcoals derive from all major plant groups, indicating that gymnosperms, pteridophytes and angiosperms all experienced increased wildfire activity at this time (see supplementary table 1 in Belcher & McElwain, 2008).

### Potential influence of superambient oxygen

During the period of evolution of the angiosperms, most models suggest that atmospheric oxygen was elevated on the order of 1.2–1.35 times current ambient levels (e.g. Bergman *et al*., 2004). This would double ignition potential in all fuel types and increase moisture of extinction by *c*. 1.4 times above that of the present day, indicating that significantly wetter fuels would have been capable of carrying a fire (Watson & Lovelock, 2013). Therefore, increased fire frequency is anticipated in all ecosystems during the early phase of angiosperm expansion. Our models considering 26% oxygen suggest that this would have doubled rates of fire spread, heat per unit area and fireline intensity in the two fuel groups capable of carrying the most intense fires (ferns and shrubs) and allowed surface fires to transition to crown fires at greater canopy base heights (see Figs 3, 4; Tables 3, 4). Therefore, increased mortality of gymnosperm trees might have been expected in the mid‐Cretaceous, due to the enhancement of crown fire potential from the coupled influence of novel fuel invasions and rising levels of atmospheric oxygen.

### Impact on conifer forests and evolutionary adaptations to fire

Many modern conifers have traits that make them resistant to fires, and the distribution of plants with these traits typically relates to the fire regime that they experience (He *et al*., 2012). Fire‐resistant traits in the Pinaceae have been shown to date back to the Cretaceous Period, with thick bark originating *c*. 126 Ma in *Pinus* and even thicker bark appearing later at *c*. 89 Ma (He *et al*., 2012). Thick bark is typical of surface fire regimes (Jackson *et al*., 1999), where it provides protection against extensive cambial heating. The evolution of this trait following the spread of angiosperms implies that surface fires must not only have existed at this time, but also supports the idea that changes in surface fire behaviour likely drove the direction of resistance trait evolution in *Pinus*. Crown fire also likely played an important role, because the evolution of serotiny in *Pinus* has been dated to 89 Ma (He *et al*., 2012), implying that changes in crown fires influenced adaptive trait evolution. Such adaptations to fire were likely to have been geographically widespread, because Cretaceous fossils of the subgenus *Pinus* are numerous throughout both America and Eurasia from mid‐ to high latitudes (Myers & Rodriguez‐Trejo, 2009). Moreover, these ecosystems evidently experienced fires because the Pinaceae are well represented in the fossil record of charcoals during this time period (Batten, 1998; Collinson *et al*., 2000; Falcon‐Lang *et al*., 2001, 2016; Glasspool & Scott, 2010).

Our model outputs explore the energy release from flaming fires, in which the majority of energy is transferred upwards. Such surface fires are capable of leading to significant tree mortality by causing heat‐induced desiccation causing canopy necrosis, by heating leaves to lethal temperatures > 60°C (Stephens & Finney, 2002). Although the amount of crown kill from scorching depends on carbon allocation within a tree, which changes with age and varies between species (Ryan & Reinhardt, 1988), in general, the best indicator of crown injury is the proportion of the crown that is scorched such that the probability of tree mortality increases as a greater volume of the crown is injured (Ryan *et al*., 1988; Stephens & Finney, 2002). Our model explorations suggest that both fern and shrub understories would have had the potential to induce canopy scorch tens of metres in to the canopy even at low mid‐flame wind speeds and had the ability to transition to crown fires in forests with a relatively low canopy base height (≤ 4 m). Therefore, the spread of angiosperm shrubs and latterly the diversification of ferns likely enhanced the occurrence of intense fires in conifer forest understories (Fig. 3b,c) and in so doing increased the risk of canopy scorch (Fig. 3e) and crown fires. Interestingly the gain of fire adaptations in *Pinus* (He *et al*., 2012) may attest to such changes where the development of even thicker bark and serotinuous cones, appears to have coincided with a shift to the more intense fires following the addition of shrub understories and the diversification of ferns.

This interpretation is consistent with the fossil record of the Cretaceous which indicates that both ferns and angiosperm surface fuels were highly flammable. There is evidence of abundant charcoalified fern remains from the Lower Cretaceous of Western Europe (Harris, 1981; Brown *et al*., 2012) and it is clear that ecosystems comprising early angiosperms did burn because some of the earliest remains of flowering plants are found as charcoal (Friis *et al*., 2006, 2011; Brown *et al*., 2012). Critically, charred conifer wood is also common in Cretaceous sediments (Brown *et al*., 2012, 2013), implying that surface fires in both fuel types were likely able to transition to crown fires in gymnosperm forests. The fossil record is however suggestive of the loss of conifer species at this time because many pinaceous fossils cannot be assigned to any modern groups (Gernandt *et al*., 2008), implying that many pinaceous taxa may have been unable to adapt to the novel fire systems that appeared during the early phases of angiosperm colonization (He *et al*., 2012). It is unlikely that fire‐induced conifer mortality would need to be widespread in order for a steady adjustment in dominance. A small but persistent doubling of the background mortality rate (such as 1–2% yr^−1^) has been shown to cause a greater than 50% reduction in mean tree age in modern forests (van Mantgem *et al*., 2009).

### The balance of conifer mortality vs angiosperm recruitment

Gymnosperm decline would occur if mortality was not compensated for by increased recruitment of gymnosperms, but instead by increased recruitment of angiosperms. Therefore, the estimated changes in fire behaviour, coupled with variations in recruitment strategies between angiosperms and gymnosperms, would need to tip the balance of post‐fire recruitment in favour of angiosperms. Crown fires readily alter the availability of light, water and nutrients in an ecosystem, which can affect the likelihood of successful conifer regeneration (Harmon, 1984). Plant species adapted to the early stages of succession, such as those that best survive or regenerate after fire, will replace species that are better able to compete for resources in the absence of fire. Angiosperms have several ecophysiological adaptations that may have facilitated their regrowth and rapid recolonization post‐fire and provided them with a competitive advantage over both ferns and gymnosperms. One ecophysiological strategy of the angiosperms is their rapid increase in leaf vein density throughout their early evolution (Brodribb & Field, 2010). High vein densities are able to support rapid water transport during transpiration, which is also linearly related to photosynthetic rate, meaning that angiosperms effectively doubled their maximum photosynthetic rate by the Late Cretaceous. However, no such alteration occurred in ferns or gymnosperms during the same period (Brodribb & Field, 2010). In addition, measurements of the stomatal conductance of angiosperms, ferns and gymnosperms grown in controlled atmospheres have shown that coordination of high vein density and high maximum stomatal conductance dramatically increases the dynamic operational conductance range of angiosperms (McElwain *et al*., 2015). Therefore, during the early evolutionary history of angiosperms, having a range of operational conductances would likely have conferred upon them greater ecophysiological plasticity, enabling angiosperms to operate within a much wider ecophysiological niche space than other seed plant groups (McElwain *et al*., 2015). If increased fire‐induced mortality created patchy and more complex landscapes, then angiosperms ought to have been better adapted to inhabit available ecological niches than either ferns or gymnosperms. Intense shrub‐ or fern‐fuelled fires could have led to fire cleared patches in forest which would have allowed invading angiosperms to reach their high photosynthetic potential in open sunlit habitats (assuming adequate nutrients, water and sufficient atmospheric CO_2_) (Bond & Scott, 2010). Interestingly, several clades of angiosperms that occur in modern fire‐prone floras appeared in the Late Cretaceous. For example, the Gondwanan Proteaceae family spent the first 25 Myr of their history in closed‐canopy forest, but moved into open fire‐prone habitats from 88 Ma, whereas the evolution of clades with fire‐cued seed release or germination strategies appeared by 71 Ma (Lamont & He, 2012). This implies that shifts in landscape heterogeneity and opening up of new ecological niches during the Late Cretaceous may have been driven by intense shrub‐ and fern‐fuelled fires that cleared areas of once dense conifer forest.

## Conclusions

Our explorations of fuel‐driven changes to Cretaceous fire behaviour suggest that the addition of angiosperm shrub understories and the diversification of ferns in the Late Cretaceous, rather than the ignition properties of weedy angiosperm fuels (e.g. Bond & Scott, 2010), most likely played a role in enabling relatively short‐stature angiosperms to outcompete tall gymnosperm trees. Using the first ever palaeofire behaviour models, we have been able to provide evidence for possible changes in fire behaviour based on the addition of novel fuel groups to the Cretaceous landscape. Postulated concomitant rising levels of atmospheric oxygen would have further increased ignitability, surface fire spread rates and fire intensity during this period. Therefore, the expansion of the angiosperms at the expense of gymnosperm trees appears to have been tied to subtle changes in fire behaviour that enhanced gymnosperm mortality, clearing patches of forest that the angiosperms were ideally suited to rapidly exploit, assisted by their suitable ecophysiological adaptations.

## Author contributions

C.M.B. designed the research; C.M.B. and V.A.H. undertook the experiments; C.M.B. undertook the modelling and the interpretation; and C.M.B. and V.A.H. wrote the manuscript.

## Supporting information

Please note: Wiley Blackwell are not responsible for the content or functionality of any Supporting Information supplied by the authors. Any queries (other than missing material) should be directed to the *New Phytologist* Central Office.

**Table S1** List of plant types tested and ignition results
**Table S2** Cretaceous fuel model parameters
**Table S3** Heat of combustion used in Cretaceous fuel models
**Methods S1** Description of the iCone Calorimeter.
