# Supplementary material for: Changes to Cretaceous surface fire behaviour influenced the spread of the early angiosperms
Source: New Phytol. 2016 Nov 7;213(3):1521–32. doi: 10.1111/nph.14264 (PMC5245107; doi:10.1111/nph.14264)
Supplement: Supplementary file 1 — Table S1 List of plant types tested and ignition results Table S2 Cretaceous fuel model parameters Table S3 Heat of combustion used in Cretaceous fuel models Methods S1 Description of the iCone Calorimeter. [file NPH-213-1521-s001.pdf]

New Phytologist

New Phytologist Supporting Information Tables S1–S3 and Methods S1

Article title: Changes to Cretaceous surface fire behaviour influenced the spread of the early angiosperms

Authors: Claire M. Belcher Victoria A. Hudspith

Article acceptance date: 3 September 2016

The following Supporting Information is available for this article:

Table S1 List of plant types tested and ignition results

Table S2 Cretaceous fuel model parameters

Table S3 Heat of combustion used in Cretaceous fuel models

Methods S1 Description of the iCone Calorimeter.

Table S1 Full ignition results from laboratory experiments that tested the flammability of analogue Cretaceous fuels

Morphotype	Species	Median time to ignition (s) fresh fuel	Median time to ignition (s) cured fuel
Angiosperm Shrub	*Hypericum* sp.	76	4.5
Angiosperm Shrub	*Buxus* sp.	85	7.5
Angiosperm Shrub	*Drimys winteri*	120	5
Angiosperm Shrub	*Laurus nobilis*	29	4
Angiosperm Shrub	*Illicium vernum*	59	6
Angiosperm Weedy	*Rubus fruticosus*	No ignition - 32 s	3
Angiosperm Weedy	*Urtica dioica*	No ignition	4
Angiosperm Weedy	*Piper nigrum*	No ignition	3
Angiosperm Weedy	*Sarcandra chloranthoides*	No ignition	4
Pteridophytes	*Asplenium scolopendrium*	No ignition	3
Pteridophytes	*Pteridium*	No ignition - 51 s	3
Pteridophytes	*Dryopteris*	No ignition	4
Pteridophytes	*Dicksonia antarctica*	51	6
Pteridophytes	*Blechnum tabulare*	No ignition	5
Pteridophytes	*Equisetum robustum*	No ignition - 35 s	6
Pteridophytes	*Equisetum* sp (very thin)	202.5	11
Gymnosperm - Needle/narrow	*Pinus radiata*	49	6
Gymnosperm - Needle/narrow	*Abies koreana*	31	10
Gymnosperm - Needle/narrow	*Sequoia sempervirens*	No ignition - 45 s	6
Gymnosperm - broad	*Cunninghamia konishii*	81	10
Gymnosperm - broad	*Podocarpus salignus*	58	6
Gymnosperm - scale leaved	*Cryptomeria japonica*	81	9
Gymnosperm - scale leaved	*Thujopsis dolabrata*	38	10
Fuel State - Leaf litter			
Pteridophyte	*Dicksonia antarctica*	9	5
Gymnosperm - Needle/narrow	*Pinus radiata*	16	7
Gymnosperm - Needle/narrow	*Sequoia sempervirens*	16	13
Gymnosperm - broad	*Cunninghamia konishii*	9	8
Gymnosperm - broad	*Podocarpus salignus*	10	8
Gymnosperm - scale leaved	*Cryptomeria japonica*	9	8
Gymnosperm - scale leaved	*Thujopsis dolabrata*	15	7

Methods S1 Description of the iCone Calorimeter.

The iCone calorimeter (Fire Testing Technology, East Grinstead, UK) is a state-of-the-art fire calorimeter. It uses a high power coiled ‘cone’ shaped heating element to deliver an evenly spread and known flux of heat to the sample. The Cretaceous analogue fuels samples were subjected to a heat flux of 50 kW m^{-2}. This is within the typical range for flammability testing (Drysdale, 2011; Tewarson, 2002) and also represents the lower range of heat fluxes experienced by virgin fuel in a wildfire (Silvani *et al*., 2009; McAllister *et al*., 2012). As the sample is exposed to the heat source, a sparking pilot igniter, placed above the sample, causes ignition of the pyrolysate gases given off by the fuel. This controlled laboratory setup, of heat and an ignition source, mimics the conditions surrounding a fuel sample when a wildfire approaches. The incoming fire heats the surface fuel, and begins to decompose the constituent plant material, first releasing water vapour (removing any moisture), followed by volatile and other thermal decomposition gases known as pyrolysate (Drysdale, 2011). Once the rate of pyrolysate release is sufficient, the nearby fire (or spark in the laboratory tests) ignites them and a flame is established on the sample. The calorimeter part of the equipment monitors the amount of oxygen depletion in a flue positioned above the burning sample. The heat released during combustion per unit mass of oxygen consumed is known to be a constant (Tewarson, 2002), and the calorimeter converts the amount of oxygen depletion caused by the fire into a measure of heat release. The iCone calorimeter was used to measure time taken for the samples to ignite (s), and the heat release rate throughout the burn duration was converted to heat of combustion per gram of fuel. The cone calorimeter is a standard piece of equipment used in fire safety and as such, tests are carried out according to the international standard, ASTM E1354 (http://www.astm.org/Standards/E1354.htm).

References

Drysdale D, ed. 2011. *An introduction to fire dynamics: 3rd edn.* Chichester, UK: John Wiley & Sons Ltd.

Tewarson A. 2002. *Generation of heat and chemical compounds in fires. SFPE Handbook of Fire Protection Engineering 3rd edn.* Quincy, MA, USA: NFPA.

Silvani X, Morandini F. 2009. Fire spread experiments in the field: temperature and heat flux measurements. *Fire Safety Journal* **44:** 279–285.

McAllister S, Grenfell I, Hadlow A, Jolly WM, Finney M, Cohen FJ. 2012. Piloted ignition of live forest fuels. *Fire Safety Journal* **51:** 133–142.

New Phytologist

Table S2 Cretaceous fuel models parameter tables

**Conifer litter - CL**	**Ambient oxygen – CL1**	**Superambient oxygen CL2**
1-h fuel load (t ha^{-1})	1.12	1.12
10-h fuel load (t ha^{-1})	4.93	4.93
100-h fuel load (t ha^{-1})	6.28	6.28
Live herbaceous fuel load (t ha^{-1})	0	0
Live woody fuel load (t ha^{-1})	0	0
1-h SA : V (m^2 m^{-3})	6562	6562
Live herbaceous SA : V (m^2 m^{-3})	5906	5906
Live woody SA : V (m^2 m^{-3})	5249	5249
Fuel bed depth (m)	0.09	0.09
Dead fuel moisture of extinction (%)	39	80
Dead fuel heat content (kJ kg^{-1})	16800	22540
Live fuel heat content (kJ kg^{-1})	16800	22540

**Fern understory FUn**	**Ambient oxygen FUn1**	**Superambient oxygen FUn2**
1-h fuel load (t ha^{-1}) (dead)	14	14
10-h fuel load (t ha^{-1}) (dead)	4	4
100-h fuel load (t ha^{-1}) (dead)	1	1
Live herbaceous fuel load (t ha^{-1})	0	0
Live woody fuel load (t ha^{-1})	1	1
1-h SA : V (m^2 m^{-3})	5741	5741
Live herbaceous SA : V (m^2 m^{-3})	4921	4921
Live woody SA : V (m^2 m^{-3})	4921	4921
Fuel bed depth (m)	0.9	S0.9
Dead fuel moisture of extinction (%)	39	80
Dead fuel heat content (kJ kg^{-1})	14180	18950
Live fuel heat content (kJ kg^{-1})	14180	18950

**Weedy understory WUn** **(based on TU3)**	**Ambient oxygen WUn1**	**Superambient oxygen WUn2**
1-h fuel load (t ha^{-1})	2.47	2.47
10-h fuel load (t ha^{-1})	0.34	0.34
100-h fuel load (t ha^{-1})	0.56	0.56
Live herbaceous fuel load (t ha^{-1})	1.46	1.46
Live woody fuel load (t ha^{-1})	2.47	2.47
1-h SA : V (m^2 m^{-3})	5906	5906
Live herbaceous SA : V (m^2 m^{-3})	5249	5249
Live woody SA : V (m^2 m^{-3})	4593	4593
Fuel bed depth (m)	0.4	0.4
Dead fuel moisture of extinction (%)	39	80
Dead fuel heat content (kJ kg^{-1})	14430	16540
Live fuel heat content (kJ kg^{-1})	14430	16540

**Shrubby understory SUn** **(based on TU5)**	**Ambient oxygen SUn1**	**Superambient oxygen SUn2**
1-h fuel load (t ha^{-1})	8.97	8.97
10-h fuel load (t ha^{-1})	8.97	8.97
100-h fuel load (t ha^{-1})	6.73	6.73
Live herbaceous fuel load (t ha^{-1})	0	0
Live woody fuel load (t ha^{-1})	6.73	6.73
1-h SA : V (m^2 m^{-3})	4921	4921
Live herbaceous SA : V (m^2 m^{-3})	5906	5906
Live woody SA : V (m^2 m^{-3})	2461	2461
Fuel bed depth (m)	0.9	0.9
Dead fuel moisture of extinction (%)	39	80
Dead fuel heat content (kJ kg^{-1})	17190	21450
Live fuel heat content (kJ kg^{-1})	17190	21450

Table S3 Heat of Combustion used in Cretaceous fuel models

**Fuel state - cured (median values) - oven dried fuel**		**iCone median mean EHoC MJ kg^{-1}**	**iCone mean**	**Bomb mean HoC MJ kg^{-1}**	**Bomb mean**	**Fuel type**	**Notes**
**Morphotype**	**Species**						
Angiosperm Shrub	*Hypericum* sp.	15.32	17.19		21.45	Angiosperm Shrubs	Taken from dry fuel because these are timber understory fuel models
	Buxus sp.	18.47		21.45			
	Drimys winteri	17.38					
	Laurus nobilis	17.38					
	Illicium vernum	17.40					
Angiosperm Weedy	*Rubus fruticosus*	14.63	14.43	17.16	16.538	Angiosperm Weedy	
	Urtica dioica	13.26		15.916			
	Piper nigrum	14.04					
	Sacandra chloranthoides	15.79					
Pteridophytes	*Hartstongue*	14.22	14.18		18.955	Pteridophytes	
	Pteridium sp.	12.71		19.76			
	Dryopteris sp.	14.62		18.15			
	Dicksonia antarctica	15.39					
	Blechnum tabulare	14.18					
	Equisetum robustum	13.90					
	Equisetum sp.	14.28					
Gymnosperm - Needle/narrow	*Pinus radiata*	14.84	16.80	22.54	22.54	Needle Leaved Conifers	Taken from dry litter because these are timber litter fuel models
	Sequoia sempervirens	16.64					
Gymnosperm - broad	*Cunninghamia konishii*	16.82				Broad Leaved Conifers	
	Podocarpus salignus	15.01					
Gymnosperm - scale leaved	*Cryptomeria japonica*	18.69				Scale Leaved Conifers	
	Thujopsis dolabrata	18.80					

EHoC, effective heat of combustion (where pyrolysis products remain, combustion is not complete; HoC, heat of combustion (combustion is complete no pyrolysis products remain, combustion is complete).
